# Luteolin inhibits GABA_A_ receptors in HEK cells and brain slices

**DOI:** 10.1038/srep27695

**Published:** 2016-06-13

**Authors:** Mei-Lin Shen, Chen-Hung Wang, Rita Yu-Tzu Chen, Ning Zhou, Shung-Te Kao, Dong Chuan Wu

**Affiliations:** 1 Graduate Institute of Chinese Medicine, China Medical University, Taichung, Taiwan; 2 Graduate Institute of Clinical Medical Science, China Medical University, Taichung, Taiwan; 3 Translational Medicine Research Center, China Medical University Hospital, Taichung, Taiwan

## Abstract

Modulation of the A type γ-aminobutyric acid receptors (GABA_A_R) is one of the major drug targets for neurological and psychological diseases. The natural flavonoid compound luteolin (2-(3,4-Dihydroxyphenyl)- 5,7-dihydroxy-4-chromenone) has been reported to have antidepressant, antinociceptive, and anxiolytic-like effects, which possibly involve the mechanisms of modulating GABA signaling. However, as yet detailed studies of the pharmacological effects of luteolin are still lacking, we investigated the effects of luteolin on recombinant and endogenous GABA_A_R-mediated current responses by electrophysiological approaches. Our results showed that luteolin inhibited GABA-mediated currents and slowed the activation kinetics of recombinant α1β2, α1β2γ2, α5β2, and α5β2γ2 receptors with different degrees of potency and efficacy. The modulatory effect of luteolin was likely dependent on the subunit composition of the receptor complex: the αβ receptors were more sensitive than the αβγ receptors. In hippocampal pyramidal neurons, luteolin significantly reduced the amplitude and slowed the rise time of miniature inhibitory postsynaptic currents (mIPSCs). However, GABA_A_R-mediated tonic currents were not significantly influenced by luteolin. These data suggested that luteolin has negative modulatory effects on both recombinant and endogenous GABA_A_Rs and inhibits phasic rather than tonic inhibition in hippocampus.

Luteolin (PubChem CID: 5280445) is a naturally occurring flavone with four additional hydroxyl groups at C3′, C4′, C5 and C7 on the flavone backbone of 2-phenylchromen-4-one (2-phenyl-1-benzopyran-4-one)^1^. Luteolin is found in many vegetables and medical herbs, such as *Perilla frutescens*^2^,^3^. The pharmacological effects of luteolin have been widely reported, such as antioxidant, anticarcinogenic, and anti-inflammatory activities^3^,^4^. Interestingly, recent studies have indicated that luteolin might have psychopharmacological effects in the central nervous system (CNS) at least partially through activation of GABA_A_Rs^5^,^6^,^7^,^8^,^9^,^10^. However, detailed studies that examine the pharmacological effects of luteolin on GABA_A_R functions are still lacking.

GABA_A_Rs are the major inhibitory receptors in the CNS. At least 19 subunits of GABA_A_Rs exist in the human nervous system, including α1-6, β1-3, γ1-3, θ, π, ε and ρ1-3^11^. The expression of GABA_A_R subunits is not limited to the nervous system but is also widely found in peripheral non-neuronal organs, including the lung, pancreas, gut etc. In the peripheral systems such as the lung tissue, endogenous GABA_A_Rs could either exist as the αβ isoform or incorporate a γ-like subunit^12^,^13^. These peripheral GABA_A_Rs are involved in various physiological and pathological conditions, including modulation of glucagon release, mucus overproduction, and prevention of cell death in pancreas and liver^13^,^14^,^15^,^16^,^17^. In the adult CNS, the major isoforms of GABA_A_Rs are composed of α, β, and γ subunits^12^,^18^. Varied compositions of GABA_A_Rs are differently distributed in synaptic and extrasynaptic regions of the inhibitory synapses to control balance between neuronal excitation and inhibition for normal brain functions. The high diversity of subunit compositions indicates that the GABA_A_R subtypes located at different brain regions and different subsynaptic loci are engaged in distinct functions^19^. For example, in the hippocampus, the α1β2γ2 GABA_A_Rs mediate the phasic inhibitory transmission at synapse, whereas the α5β2γ2 GABA_A_Rs are located at extrasynaptic sites to mediate tonic inhibition in response to ambient release of GABA^12^. Previous studies showed that knockout α5 subunit of GABA_A_Rs or selective inhibition of α5β2γ2 function improved learning and memory abilities^20^,^21^, while enhancement of synaptic GABA_A_R function has always been an important target of treating epilepsy, anxiety, and other psychiatric/neurological diseases^12^.

Despite accumulating studies suggesting a modulatory effect of luteolin on GABA_A_Rs in the CNS^7^,^9^, the pharmacological characterization of luteolin still remains unclear. For instance, studies have shown that luteolin has antidepressant and analgesic effects that are considered to be mediated by enhancing GABA_A_R functions^6^,^8^; however, luteolin did not exhibit any pro- or anti-convulsant effects in various animal models of epilepsy^22^. Furthermore, luteolin has been reported to enhance learning and memory in a neurodegenerative model^10^. Children with autism spectrum disorders showed improvement in adaptive functioning after receiving dietary supplement of flavonoids including luteolin (100 mg/10 kg weight daily)^23^, which produced an estimated concentration of ~8.7 μmol/L in plasma according to a study on the relationship between oral intake and plasma concentration of luteolin^24^. Hence, these previous studies have led us to hypothesize that luteolin might have varied effects on different subtypes of GABA_A_Rs to achieve the complex neuropharmacological influences. To elucidate the pharmacological mechanisms underlying these study results, we investigated the sensitivities of α1- and α5-containing GABA_A_Rs to different doses of luteolin in HEK cells. Furthermore, the effect of luteolin on α1- and α5- containing GABA_A_R- mediated phasic and tonic inhibition in hippocampal slices was also studied.

## Results

### Effects of luteolin on dose-response relationships of GABA_A_Rs in HEK cells

Inclusion of α and β subunits is required to create functional GABA_A_R pentamers and the αβ receptors are already sufficient for insertion into the plasma membrane. Despite low levels of expression, the αβ receptors naturally exist in the CNS and in peripheral systems^13^,^25^. The αβ receptors are also useful for molecular pharmacology studies using recombinant expression systems like HEK cells. The αβγ receptors are the most abundant form of GABA_A_Rs in the CNS and are sensitive to benzodiazepine potentiation. Among the αβγ compositions, the α1β2γ2 receptors are the most predominant form and are ubiquitously distributed in inhibitory synapses to mediate phasic inhibition, whereas α5β2γ2 receptors are predominantly located at the extrasynaptic region to mediate the tonic inhibition in hippocampus. In the present study, we selected recombinant α1β2, α1β2γ2, α5β2, and α5β2γ2 GABA_A_Rs to characterize the pharmacological effects of luteolin.

**Figure 1:**
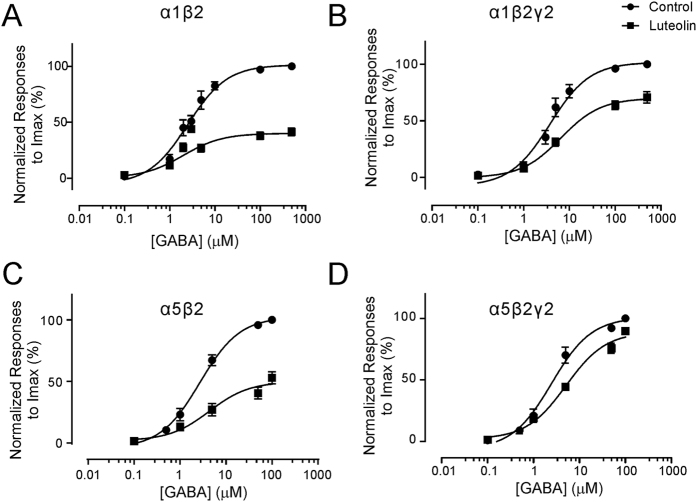
Inhibition of GABA-activated currents by luteolin in the recombinant GABA_A_Rs. Recombinant GABAARs composed by α1β2 (**A**), α1β2γ2 (**B**), α5β2 (**C**), and α5β2γ2 (**D**) were first activated by GABA in the absence of luteolin (circles). Dose-response relationships were calculated from the value of peak whole-cell current amplitudes induced by varying GABA concentrations normalized to the value of I_max_ that were activated by maximum GABA concentration. In the presence of 50 μM of luteolin (square), the amplitudes of GABA-activated currents were also normalized to the value of I_max_ of the same cell. All data points and bars represent mean values ± s.e.m. More detailed data for the dose-response curves of GABAARs are presented in Table 1.

Whole-cell recordings were performed in HEK293T cells expressed with α1β2, α1β2γ2, α5β2, or α5β2γ2 subunits of GABA_A_Rs. Fast-perfusion of GABA (0.1–500 μM) produced inward current responses at a holding membrane potential of −60 mV. Recombinant expression of GABA_A_Rs resulted in dose-response curves with EC_50_ values within a range of 2 to 4 μM (Fig. 1, Table 1). Extracellular application of luteolin (50 μM) inhibited current responses induced by the agonist from medium to saturating doses (1–500 μM GABA) in all tested forms of GABA_A_Rs. Although the maximum currents (I_max_) of GABA_A_Rs were substantially reduced by luteolin, the EC_50_ was relatively less affected (with 0.8-, 1.7-, 1.6-, and 2.1-fold changes by 50 μM luteolin in α1β2, α1β2γ2, α5β2, and α5β2γ2 receptors, respectively, Table 1). These results indicate that luteolin is likely a non-competitive antagonist of GABA_A_Rs.

### Potency and efficacy of luteolin on different forms of GABA_A_Rs

**Figure 2:**
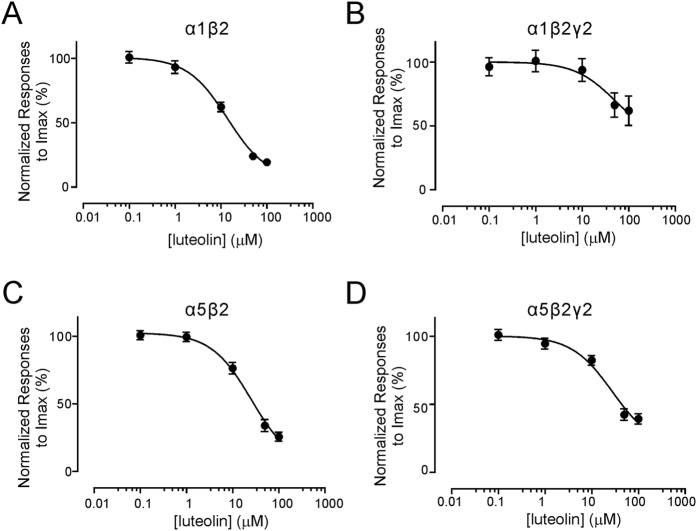
Inhibition curves of luteolin in the recombinant GABA_A_Rs. GABA currents were activated by 3 μM of GABA in α1β2 (n = 8) (**A**) and α1β2γ2 (n = 10) (**B**) receptors, and by 2 μM of GABA in α5β2 (n = 10) (**C**) and α5β2γ2 receptors (n = 10) (**D**). Inhibition curves were calculated by normalizing values of the relative currents obtained following application of varying concentrations of luteolin to the values obtained in the absence of luteolin. All data points and bars represent mean values ± s.e.m.

We next examined the potency and efficacy of luteolin on the four forms of GABA_A_Rs by testing the dose-response relationship of luteolin-mediated inhibition. 0.1–100 μM of luteolin was included in the perfusion solution and was applied onto the same cell in sequence. By using medium doses of GABA to induce current responses, luteolin strongly inhibited GABA currents at concentrations>=10 μM (Fig. 2), which exhibited a similar pattern to the effects of other flavonoids like apigenin and quercetin on GABA_A_Rs^26^,^27^. By comparing the IC_50_ of luteolin on the four types of GABA_A_Rs, we found that luteolin inhibited GABA currents with similar potency, with the lowest IC_50_ in α1β2 (10.8 ± 4.46 μM). In the other forms, the IC_50_ values of luteolin are 51.4 ± 242.8 μM in α1β2γ2, 25.4 ± 9.0 μM in α5β2, and 27.5 ± 13.8 μM in α5β2γ2. Moreover, the inhibitory efficacy of luteolin was assessed by the extent of reduction in I_max_. Our results showed that luteolin had better efficacy on α1β2 and α5β2 receptors (Table 1). Inclusion of the γ2 subunit decreased the efficacy of luteolin, suggesting that the γ2 subunit is not required for forming luteolin binding site for its inhibition.

**Figure 3:**
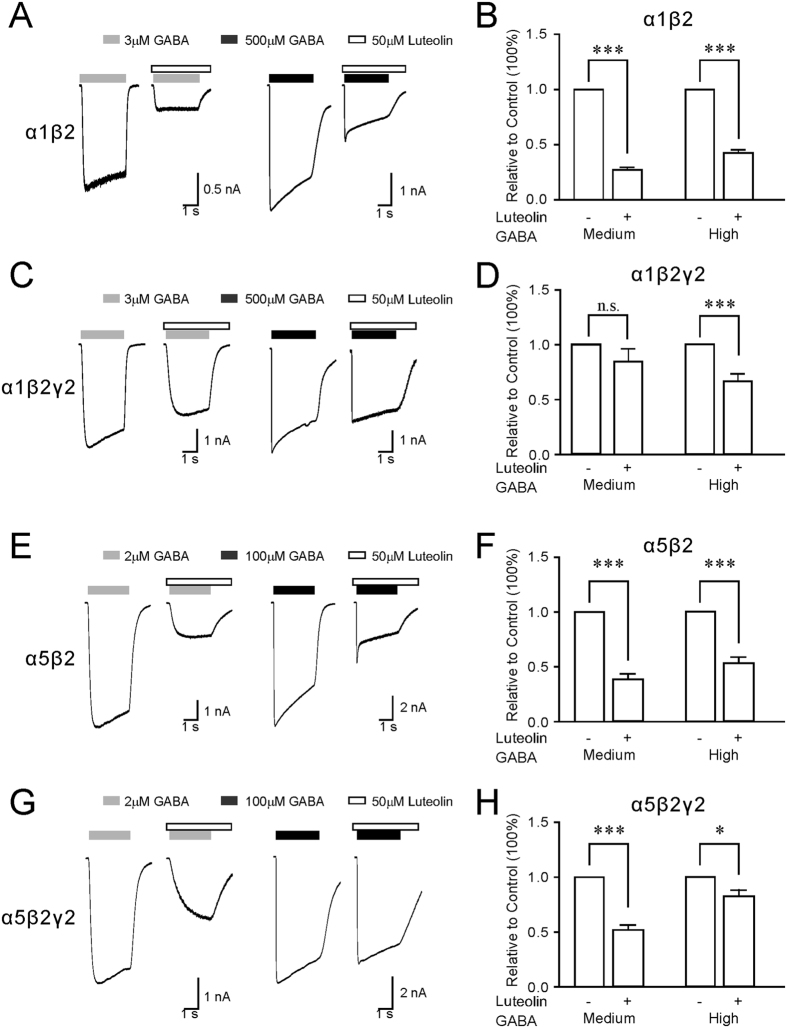
Inhibition effect of luteolin on medium versus high dose of GABA-activated current responses in recombinant GABA_A_Rs. Representative current traces showed medium or high doses of GABA-activated current responses in α1β2 (**A**), α1β2γ2 (**C**), α5β2 (**E**), and α5β2γ2 receptors (**G**) before and after 50 μM of luteolin. The quantitative results of luteolin inhibition were calculated from the value of GABA currents in the presence of luteolin normalized to the value before luteolin in α1β2 (n = 9) (**B**), α1β2γ2 (n = 9) (**D**), α5β2 (n = 10) (**F**), and α5β2γ2 receptors (n = 10) (**H**). All data points and bars represent mean values ± s.e.m. *P < 0.05; **P < 0.01; ***P < 0.001; n.s., no significance using student’s t-test.

We also compared the inhibitory efficacy of luteolin on current responses mediated by medium or high doses of GABA. In the α1β2 and α5β2 receptors, both medium- and high-dose GABA-mediated currents were significantly inhibited by 50 μM of luteolin (Fig. 3A,C). In the α1β2γ2 receptors, however, luteolin had an inhibitory effect on 500 μM but not 3 μM GABA-induced currents (Fig. 3B). In the α5β2γ2 receptors, luteolin showed a better efficacy on 2 μM compared with 500 μM GABA-mediated currents (Fig. 3D). Taken together, these data showed that luteolin exhibited varied degrees of potency and efficacy to reduce current responses of different forms of GABA_A_Rs.

### Luteolin affected activation kinetics of GABA_A_Rs

**Figure 4:**
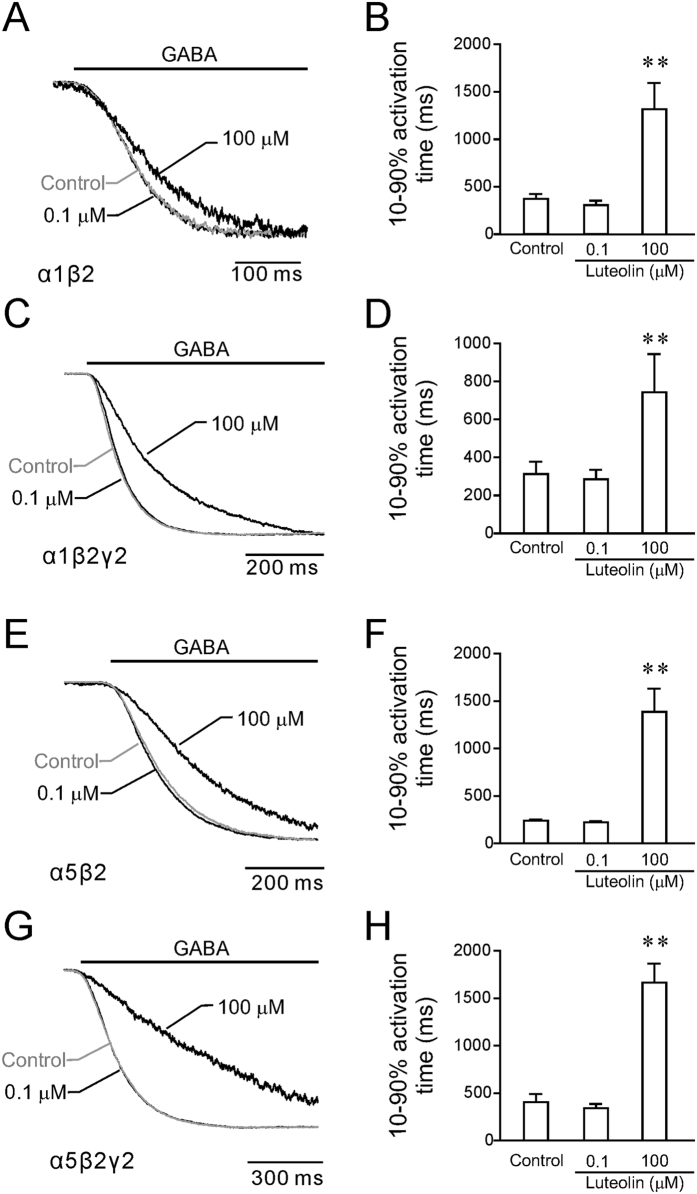
Luteolin slowed the activation of recombinant GABA_A_Rs. Representative current traces (superimposed and scaled) illustrating the effect of 0.1 and 100 μM of luteolin on current activation of α1β2 (**A**), α1β2γ2 (**C**), α5β2 (**E**), and α5β2γ2 receptors (**G**). The quantitative results summarized the 10–90% activation time calculated for control currents or after 0.1 and 100 μM of luteolin in α1β2 (n = 15) (B), α1β2γ2 (n = 10) (**D**), α5β2 (n = 10) (**F**), and α5β2γ2 receptors (n = 10). All data points and bars represent mean values ± s.e.m. **P < 0.01compared with control using one-way ANOVA.

Activation kinetics of GABA currents can influence the time course of the neurotransmitter-evoked responses at the synapses^28^. In the present experiments, currents were elicited by fast perfusion of GABA at concentrations close to EC_50_, and the activation kinetics were characterized by the 10–90% activation time of the current after fast perfusion of GABA. Luteolin at high concentrations (100 μM) slowed the activation time in all tested forms of GABA_A_Rs (Fig. 4). In the presence of 100 μM luteolin, the activation time of α1β2, α1β2γ2, α5β2, and α5β2γ2 GABA_A_Rs after luteolin treatment was increased by 3.5-, 2.4-, 5.8-, and 4.1-fold, respectively. Luteolin concentration lower than 10 μM had no significant effects on GABA_A_R activation time. Here we selected 0.1 and 100 μM as the representative doses of luteolin to show their effects on the activation kinetics of GABA currents. Our data suggested that higher doses of luteolin prolonged activation time of GABA_A_Rs (Fig. 4).

### Effects of luteolin on phasic currents mediated by GABA_A_Rs in hippocampal slices

**Figure 5:**
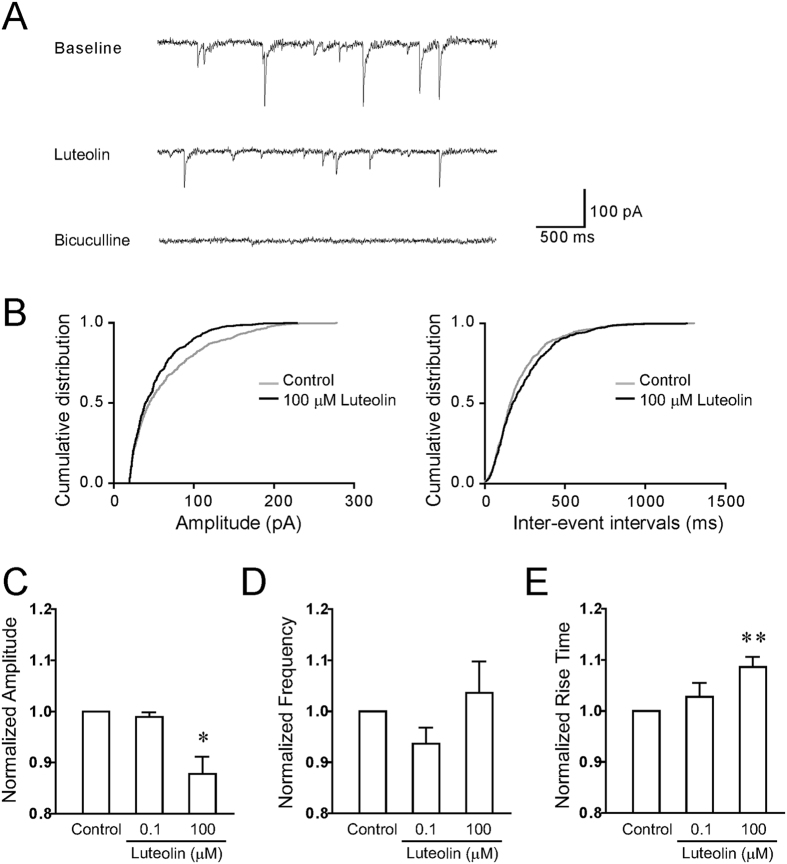
The effect of luteolin on mIPSCs in hippocampal slices. (**A**) Representative traces showed mIPSCs recorded before (top trace) or after 100 μM of luteolin treatment (middle trace) in a hippocampal CA1 pyramidal neuron. 10 μM of bicuculline was applied at the end of the experiment to eliminate mIPSCs (bottom trace). (B) Cumulative probability plots of mIPSC amplitudes (left) and inter-event intervals (right) from the recorded neuron shown in (**A**). (**C–E**) Summary of changes in mean mIPSC amplitudes (**C**), mean frequencies (**D**), and mean rise time (**E**) after 0.1 (n = 7) or 100 μM (n = 9) luteolin treatments. All data points and bars represent mean values ± s.e.m. *P < 0.05, **P < 0.01compared with control using one-way ANOVA.

The hippocampus is the critical locus for learning and memory. The α1β2γ2 GABA_A_Rs are located at the postsynaptic site of inhibitory synapses and predominantly mediate the fast inhibitory synaptic currents in hippocampus. To further investigate the pharmacological modulation of luteolin on endogenous α1β2γ2 GABA_A_Rs under physiological conditions, we tested the effect of luteolin on phasic inhibition in hippocampal slices. The GABA_A_R-mediated mIPSCs were pharmacologically isolated by inclusion of the sodium channel blocker TTX (0.5 μM) and the AMPA/kainate receptor blocker CNQX (20 μM). At the end of each experiment, the GABA_A_R antagonist bicuculline (10 μM) was applied to confirm that the mIPSCs were abolished (Fig. 5A). Here we selected two representative doses (0.1 and 100 μM) to test the effect of luteolin. We showed that 100 μM luteolin significantly decreased the amplitude (87.7 ± 3.4% normalized to baseline) but not the frequency (103.6 ± 6.2%) of mIPSCs (Fig. 5A–D). The rise time of mIPSCs was also significantly slower after high dose of luteolin treatments (108.6 ± 2.0%) (Fig. 5E). In contrast, 0.1 μM luteolin did not show any significance in changing the frequency (93.7 ± 3.1%), amplitude (98.9 ± 0.9%), or the rise time (102.7 ± 2.7%) of mIPSCs (Fig. 5C–E). Together, these data indicated that luteolin exerts negative modulation on phasic inhibitory responses by reducing the amplitude and slowing down the activation time of synaptic currents.

### Effects of luteolin on tonic currents mediated by GABA_A_Rs in hippocampal slices

**Figure 6:**
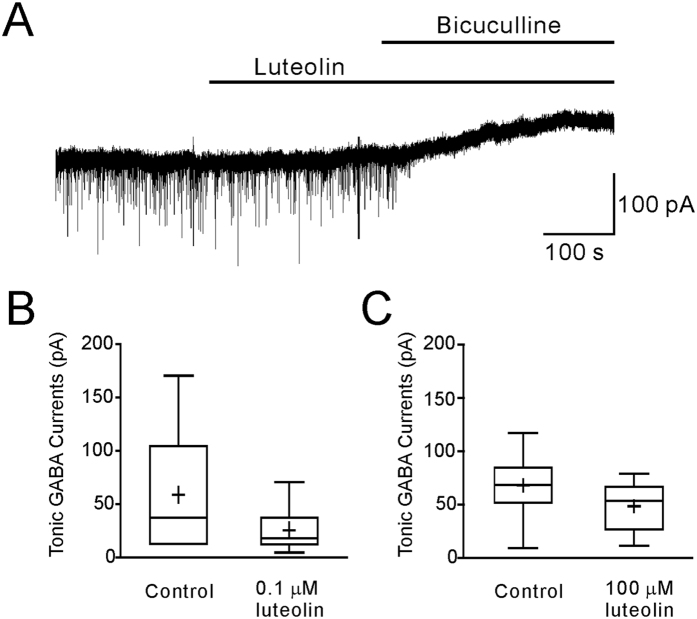
The effect of luteolin on tonic inhibitory currents in hippocampal slices. (**A**) The representative recording showed the tonic currents before and after 100 μM of luteolin treatments in a CA1 pyramidal neuron. The tonic current was revealed by 10 μM bicuculline at the end of the experiment. (**B,C**) Whisker plots (boxes, 25–75%, whiskers, Min-Max; lines, median; + , mean) showed that 0.1 μM (n = 6) (**B**) or 100 μM of luteolin (n = 10) (**C**) had no significant effects on tonic inhibition by using student’s t-test.

The α5β2γ2 GABA_A_Rs are located at the extrasynaptic site and mediate tonic inhibition in hippocampus. Our data have shown that, in the recombinant α5β2γ2 GABA_A_Rs, luteolin inhibited medium to high doses GABA-mediated responses. Considering that tonic inhibitory currents in the CNS are ascribed to the low concentration of ambient GABA in the extracellular space (from 0.2 to 2.5 μM)^29^, it still remained to be determined whether luteolin has any modulatory effect on the low-dose GABA-mediated responses in slices. In our experiments, tonic currents were defined as the shift of holding currents after application bicuculline (10 μM). In some experiments, we also included a low dose of GABA in the bath solution (0.5 μM) to unify the ambient GABA concentration. Treatments with 0.1 or 100 μM luteolin did not significantly affect the amplitudes of tonic currents (P > 0.05 for both 0.1 and 100 μM groups) (Fig. 6). These results suggested that luteolin did not influence extrasynaptic GABA_A_R-mediated tonic inhibitory currents.

## Discussion

### The pharmacological effect of luteolin on recombinant and endogenous GABA_A_Rs

Luteolin has been widely studied for its pharmacological effects, including anti-inflammatory, anti-oxidant, and anticarcinogenic activities. Recent studies have suggested that luteolin might enhance the function of GABA_A_Rs, thereby producing antihyperalgesic, anxiolytic, and antidepressant-like effects in the CNS^5^,^6^,^8^. However, it still remains unclear whether luteolin takes these effects by directly targeting on GABA_A_Rs. In the brain, modulation of distinct subunit compositions of GABA_A_Rs is associated with different neurological and behavioral outcomes. Enhancements of α1β2γ2 GABA_A_Rs that mainly mediate fast synaptic inhibition generally produce sedative effects, while inhibition of α5β2γ2 GABA_A_Rs by either pharmacological or transgenic approaches can promote learning and memory. In the present study, we showed that low concentration of luteolin (0.1 μM) did not affect either GABA_A_R-mediated phasic or tonic currents. In contrast, high concentration of luteolin (100 μM) reduced the amplitude of mIPSCs and prolonged the activation time course, but had no effect on mIPSC frequency. This indicated that the effect of luteolin was likely due to postsynaptic rather than presynaptic modulation. The inhibition of mIPSCs by luteolin was consistent with the observation from HEK cells: high concentration of luteolin suppressed the amplitude and prolonged the activation kinetics in recombinant GABA_A_Rs including the α1β2γ2 form. Although we have shown that luteolin reduced current responses that were induced by high dose but not medium dose of GABA in recombinant α1β2γ2 receptors (Fig. 3D), it was still in line with the results from slices since vesicular release of GABA at synapses normally reaches a very high concentration^30^. Furthermore, our results are consistent with previous studies that luteolin did not affect the threshold of seizure induction by pilocarpine or electrical stimuli in animal models^22^, suggesting that luteolin cannot produce obvious sedative effect through enhancement of the function of α1β2γ2 GABA_A_Rs.

The tonic inhibition in hippocampus was unaffected by luteolin even at concentrations as high as 100 μM. However, the α5β2γ2 GABA_A_R-mediated responses in HEK cells were significantly reduced. This could be ascribed to the low concentration of ambient GABA in the extracellular space in hippocampal slices, since luteolin showed very weak efficacy to modulate low dose of GABA-mediated responses (at [GABA] < 1 μM) (Fig. 1D). Therefore, our findings indicated that luteolin is a negative modulator for GABA_A_Rs and did not have any potentiation effect on phasic or tonic inhibition in hippocampus.

Previous studies of luteolin mostly focused on the *in vivo* effects. One of the major differences between the *in vivo* and *in vitro* environments is the temperature. In our study, we performed *in vitro* electrophysiological experiments in room temperature (23–25 °C), which was lower than the body temperature (37 °C). Notably, some allosteric modulators, such as zolpidem, can modulate GABA_A_Rs in a temperature-dependent manner^31^. The affinity of zolpidem to GABA_A_Rs increased along with the increasing temperature from 16, 26 to 36 °C^31^. Previous studies showed that luteolin was stable at 37 °C in culture medium for 24 hours^32^. We thereby predict that luteolin might consistently take effects and show increased inhibition on GABA_A_Rs *in vivo*. Moreover, our data revealed that luteolin showed stronger inhibitory effects on recombinant GABA_A_Rs in HEK cells than in the endogenous GABA_A_Rs in brain slices. It was possibly due to the lack of synaptic scaffolding proteins in HEK cells and this might affect luteolin-mediated inhibition on GABA_A_Rs as suggested by previous studies^33^.

### Luteolin likely targets at non-benzodiazepine binding sites of GABA_A_Rs

Benzodiazepines are one of the most potent positive modulators of γ-containing GABA_A_Rs and the binding site is located at the α(+)/γ(−) interface. Previous studies have indicated the structural similarity between flavones and benzodiazepine ligands^34^. Among different types of flavones, the presence of electronegative groups at the C6 and C3′-position are critical determinants for high affinity to the benzodiazepine-binding site^35^. For instance, hispidulin (4′,5,7-trihydroxy-6-methoxyflavone) bearing a methoxyl group at C6 is a potent benzodiazepine site ligand and potentiates GABA_A_R-mediated responses in α1β2γ2 form but not in α1β2 form receptors^36^. The structure of luteolin (5,7,3′,4′-Tetrahydroxyfavone) is similar to apigenin (5,7,4′-Trihydroxyflavone) and quercetin (3,5,7,3′,4′-Pentahydroxyfavone), all of which lack electronegative moieties at C6. Previous studies have demonstrated that luteolin and quercetin have weak affinities for the benzodiazepine site, with K_i_ values over 100 μM for the [3H]flunitrazepam binding competition^35^. Apigenin exhibited a higher affinity for central benzodiazepine receptors (with a K_i_ of 4 μM to compete [3H]flunitrazepam binding)^37^ and exerted anxiolytic and antidepressant effects in *in vivo* animal models. However, whether a direct involvement of GABA_A_Rs in the CNS is responsible of the effects of apigenin remains questionable. Electrophysiological studies showed that apigenin and quercetin similarly inhibited GABA-induced currents, while the inhibition of apigenin on α1β2γ2 GABA_A_R-mediated responses were not prevented by the benzodiazepine site antagonist flumazenil^26^. These studies indicated that, different from the traditional anxiolytic chemical benzodiazepine, the CNS effects of apigenin and quercetin are not likely due to their direct interaction and potentiation of GABA_A_Rs. In the present study, we found that luteolin negatively modulated GABA_A_Rs that lacked γ subunits. In agreement with previous studies using the [3H]flunitrazepam binding assay, our results indicated that luteolin inhibited GABA_A_Rs through non-benzodiazepine site of GABA_A_Rs. Such a modulatory effect of luteolin is in resemblance of that of apigenin and quercetin. We did not observe apparent potentiation effects of luteolin on either α1- or α5- containing GABA_A_Rs that were reported for hispidulin, likely due to the lack of a hydroxyl group at the C6 position of flavone^9^. Therefore, although we did not exclude the possible interaction between benzodiazepine sites and luteolin’s metabolites, the direct effect of luteolin on GABA_A_Rs did not involve binding to central benzodiazepine receptors. In other words, the CNS effects of luteolin are likely obtained through other pharmacological mechanisms, possibly similar to those of apigenin and quercetin due to their structural similarity.

### Different modulation on αβ and αβγ forms of GABA_A_Rs indicated possible luteolin-binding sites

During the development of benzodiazepine ligands, researchers have discovered new allosteric modulation sites on GABA_A_Rs. Ramerstorfer *et al*. have revealed a new ligand-binding site at the α(+)/β(−) interface that was independent from the benzodiazepine site at the α(+)/γ(−) interface^38^. This new binding site was discovered by screening of benzodiazepine site ligand and was determined by CGS 9895 that could potentiate GABA_A_Rs in a flumazenil insensitive manner regardless of the incorporation of γ subunits, indicating that CGS 9895 targets on non-BZ-binding sites of GABA_A_Rs^38^. Interestingly, CGS 9896, which is a structural analog of CGS 9895, exhibited a similar manner to 6-Methylflavone in a pharmacophore model of benzodiazepine site binding^34^. Given that the inhibition by luteolin, apigenin, and quercetin is independent from γ incorporation and is insensitive to flumazenil, it is reasonable to speculate the possibility that these flavones might interact with the newly identified CGS 9895-binding site at the α(+)/β(−) interface. Our results showed that luteolin had more potent effects on the αβ compared with the αβγ receptors, agreeing with the fact that the αβ receptors embrace two α(+)/β(−) interfaces while the αβγ form only contains one α(+)/β(−) interface. However, the limited information about the exact molecular location of CGS 9895-binding site precludes further investigation of this hypothesis. In addition, we cannot rule out the possibility that luteolin targets on other modulation sites like the neurosteroid-binding site, which is located at the transmembrane domains of GABA_A_Rs.

## Methods

### cDNA constructs and transfection

Rat GABA_A_R α1, α5, β2, and γ2 subunits were subcloned into the pCDNA3.1 expression vector. HEK293T cells (6 × 10^5^) were transfected with purified plasmids encoding GABA_A_Rs (total plasmid amount 2–2.5 μg) by electroporation (NEPA21, NEPA GENE). A small amount (0.2 μg) of pcDNA3-GFP was co-transfected along with GABA_A_Rs to act as a transfection marker and facilitate the visualization of transfected cells during electrophysiological experiments. After transfection, cells were re-plated on poly-(D-lysine)-coated glass coverslips and were grown in DMEM in 24-well plates for 16–24 h before patch-clamp recordings.

### Slice preparation

Hippocampal slices were prepared from 12–21-day-old ICR mice. Treatments of animals were evaluated and approved by the Institutional Animal Care and Use Committee of China Medical University according to Care of the animals and surgical procedures of China Medical University Protocols. Mice were anaesthetized with Urethane and decapitated. Brains were removed into ice-cold slicing solution containing (in mM): 230 sucrose, 26 NaHCO_3_, 10 D-glucose, 2.5 KCl, 1.25 NaH_2_PO_4_, 0.5 CaCl_2_, and 10 MgSO_4_. 350 μm-thick transverse hemi-sections from hippocampus were sliced (Leica vibratome) in the slicing solution. Then the slices were transferred to a storage chamber or the recording chamber with fresh artificial cerebrospinal fluid (ACSF) containing the following (in mM): 128 NaCl, 2.5 KCl, 2.0 MgCl_2_, 2.0 CaCl_2_, 1.25 NaH_2_PO_4_, 26 NaHCO_3_, and 10 D-glucose, and were incubated at room temperature for >1 h before recording. All solutions were saturated with 95% O_2_/5% CO_2_.

### Electrophysiology

For recording HEK cells, whole-cell patch clamp recordings were performed under voltage-clamp mode using the AXOPATCH 200B amplifier (Molecular Devices, USA). Whole-cell currents were recorded with a holding potential of −60 mV and signals were acquired via a Digidata 1440A analog-to-digital interface and were low-pass filtered at 2 kHz and digitized at 10 kHz. Patch electrodes (3–7 MΩ) were pulled from 1.5 mm outer diameter thin-walled glass capillaries in three stages on a Flaming-Brown micropipette puller and were filled with intracellular solutions (ICS), which contained (in mM) 140 CsCl, 10 HEPES, 4 Mg-ATP and 0.5 BAPTA (pH 7.20, osmolarity, 290–295 mOsm). The coverslips were continuously superfused with the extracellular solution containing (in mM): 140 NaCl, 5.4 KCl, 10 HEPES, 1.0 MgCl_2_, 1.3 CaCl_2_ and 20 glucose (pH 7.4, 305-315 mOsm). To evoke GABA currents, we used fast perfusion of GABA with a computer-controlled multibarrel fast perfusion system (Warner Instruments, CT, USA). For recording hippocampal neurons, slices were perfused with ACSF. Hippocampal CA1 pyramidal neurons were recorded with whole-cell patch clamp with a holding potential of −60 mV under voltage-clamp model using the MultiClamp 700B amplifier (Molecular Devices, USA). Recording pipettes (3–7 MΩ) were filled with the ICS that was mentioned above. For testing the effects of luteolin, slices were pretreated with luteolin for 5–10 min. For recording miniature inhibitory postsynaptic currents (IPSCs), all bath solutions (ACSF) contained 0.5 μM TTX and 20 μM CNQX. The seal tests were performed through the application of a −5 mV step about every 5 min to monitor the changes in access resistance. Data were collected only if the whole-cell access resistance was consistent throughout the recording (changes  < 15%). All experiments were performed at 23–25 °C.

### Data analysis

Values are expressed as mean ± s.e.m. One-way ANOVA or a two-tailed Student’s t-test was used for statistical analysis and P values less than 0.05 were considered to be statistically significant. Peak current amplitude and 10–90% rises time of recombinant GABA_A_R-mediated response was measured by Clmapfit 10. Maximum currents (I_max_) were determined as the amplitude of peak currents induced by saturated concentration or indicated concentration of agonists. Dose-response curves were created by fitting data to the Hill equation: I = I_max_/[1 + (EC_50_/[A])^nH^], where I is the current, [A] is a given concentration of agonist and n_H_ is the Hill coefficient (GraphPad Prism 6, CA, USA). The amplitude, frequency, and rise time of mIPSCs were measured by MiniAnalysis. The amplitude of tonic currents was revealed by measuring the change in the holding current evoked by applying the GABA_A_R antagonist bicuculline. The baseline current of 10 s was selected for each treatment and was analyzed by generating an all-points histogram and fitting a Gaussian distribution to the positive side of the histogram by Clampfit 10. The means of the fitted Gaussian were used to determine the holding current before and after drug application^39^.

## Additional Information

**How to cite this article**: Shen, M.-L. *et al*. Luteolin inhibits GABA_A_ receptors in HEK cells and brain slices. *Sci. Rep.* **6**, 27695; doi: 10.1038/srep27695 (2016).

## Figures and Tables

**Table 1:** Data of dose-response relationship for recombinant GABA_A_Rs before and after luteolin (50 μM) treatment.

Subunit Composition	Treatment	EC_50_ (μM)	Hill Coefficient	I_max_ Normalized to Control (%)	n
α1β2	Control	2.72 ± 0.35	1.31	100	14
	Luteolin	2.00 ± 0.75	1.10	42.4 ± 3.2	14
α1β2γ2	Control	4.18 ± 0.60	1.55	100	12
	Luteolin	6.62 ± 2.11	0.97	66.5 ± 6.8	12
α5β2	Control	2.71 ± 0.42	1.19	100	9
	Luteolin	4.34 ± 2.52	0.72	53.0 ± 5.5	9
α5β2γ2	Control	2.48 ± 0.51	1.37	100	10
	Luteolin	5.10 ± 0.98	0.39	82.4 ± 5.6	10

Data from whole-cell recordings made from GABA_A_Rs expressed in HEK293T cells. I_max_, maximum currents. Data represent the mean ± S.E.M.
